# Cholesterol-Related lncRNAs as Response Predictors of Atorvastatin Treatment in Chilean Hypercholesterolemic Patients: A Pilot Study

**DOI:** 10.3390/biomedicines11030742

**Published:** 2023-03-01

**Authors:** Isis Paez, Yalena Prado, Pía Loren, Carmen G. Ubilla, Nelia Rodríguez, Luis A. Salazar

**Affiliations:** Center of Molecular Biology and Pharmacogenetics, Department of Basic Sciences, Faculty of Medicine, Universidad de La Frontera, Temuco 4811230, Chile

**Keywords:** lncRNAs, atorvastatin, normal cholesterol, hypercholesterolemia, THP-1

## Abstract

Statins are currently the treatment of choice for hypercholesterolemia. However, wide interindividual variability has been observed in the response to treatment. Recent studies have reported the role of lncRNAs in the metabolism of lipids; nevertheless, there are few studies to date that show their role in the response to treatment with statins. Thus, the aim of this study was to assess the levels of expression of three lncRNAs (RP1-13D10.2; MANTIS; lncHR1) associated with genes involved in cholesterol homeostasis in leukocyte cells of hypercholesterolemic patients after treatment with atorvastatin and compare them with levels in subjects with normal cholesterol levels. A secondary aim was to assess the levels of expression in monocytic THP-1 cells differentiated to macrophages. The study included 20 subjects with normal cholesterol (NC) levels and 20 individuals with hypercholesterolemia (HC). The HC patients were treated with atorvastatin (20 mg/day/4 weeks). THP-1 cells were differentiated to macrophages with PMA and treated with different doses of atorvastatin for 24 h. Expression of lncRNAs was determined by RT-qPCR. The lncRNAs RP1-13D10.2 (*p* < 0.0001), MANTIS (*p* = 0.0013) and lncHR1 (*p* < 0.0001) presented increased expression in HC subjects compared with NC subjects. Furthermore, atorvastatin had a negative regulatory effect on the expression of lncHR1 (*p* < 0.0001) in HC subjects after treatment. In vitro, all the lncRNAs showed significant differences in expression after atorvastatin treatment. Our findings show that the lncRNAs tested present differential expression in HC patients and play a role in the variability reported in the response to atorvastatin treatment. Further research is needed to clarify the biological impact of these lncRNAs on cholesterol homeostasis and treatment with statins.

## 1. Introduction

Long non-coding RNAs (lncRNAs) are defined as long RNA transcripts containing more than 200 nucleotides that cannot be translated into proteins [1]. The role of lncRNAs in lipid homeostasis has gradually been elucidated over recent years. Recent studies have reported the role of lncRNAs in the metabolism of lipids through their effects on SREBP transcription factors, apolipoproteins, the metabolism of triglycerides, and cholesterol uptake from macrophages and efflux [2,3,4,5]. Muret et al. provide the most exhaustive list of the lncRNAs involved in lipid metabolism, identifying 60 genes; they identify the action mechanisms of 25 of these [6].

In recent decades, the use of hypolipidemic drugs like HMG-CoA reductase inhibitors (statins) has become widespread due to their proven effectiveness in the management of dyslipidemia [7,8], producing a reduction of up to 30% in mortality attributable to cardiovascular disease [9]. Nevertheless, despite the many benefits obtained from the use of statins, differences have been observed in the responses of patients treated with the same dose of statins [10,11]. 

Recently, PCSK9 therapy is a welcome treatment option for statin intolerant patients who require treatment of their hyperlipidemia. PCSK9 inhibitors are especially beneficial in the treatment of familial hypercholesterolemic patients who are intolerant to statins or have an elevated LDL-C level despite being on maximally tolerated statin therapy. Intuitively, addition of a PCSK9 inhibitor to low dose statin therapy will be more effective in lowering LDL and avoiding the side effects of statins, since low dose and high dose statin regimens have yielded similar efficacy when combined with PCSK9 inhibitors [12]. Nevertheless, to date statins are the most widely used drugs for the treatment of hypercholesterolemia. 

Few studies to date have shown the role of lncRNAs in determining interindividual variation in the response to hypolipidemic drugs like statins. Mitchel and collaborators recently showed increased expression of the lncRNA RP1-13D10.2 in response to treatment with statin, while in the absence of statins or after the silencing of SREBP2, expression of the same lncRNA diminished [13]. After treatment with atorvastatin, the expression of the lncRNAs LASER and PCSK9 increased simultaneously both in the peripheral blood mononuclear cells (PBMC) of patients with coronary artery disease (CAD), and in HepG2 cells in vitro [14]. The lncRNA MANTIS is also strongly regulated by laminar flow and statins, through mechanisms involving the transcription factors KLF2 and KLF4 [15]. Huang and collaborators reported that atorvastatin improves the therapeutic effectiveness of exosomes derived from mesenchymal stem cells (MSC) in acute myocardial infarction through regulation of the lncRNA H19 [16].

It is interesting to note that the lncRNA NEXN-AS1 (Nexilin F-actin binding protein antisense RNA 1) is closely linked with the response to treatment with statins. Atorvastatin significantly induced the expression of NEXN-AS1 and NEXN, suggesting a new atheroprotective mechanism for the non-lipid-reducing effects of statins [17]. The lincRNA OLMALINC (oligodendrocyte maturation-associated long intergenic non-coding RNA) was recently described as a novel epigenetic regulator of serum triglyceride and of stearoyl-coenzyme A desaturase (SCD) in non-alcoholic fatty liver disease (NAFLD). Nineteen genes of the human liver co-expression network were observed, including key genes of the cholesterol path and several new statin response genes, among them the lincRNA OLMALINC. Patients who used statins showed greater hepatic expression of OLMALINC than those not treated with the drug [18].

One of the studies with the widest cover of this area is the work of Jiang and collaborators, who studied the regulation of lncRNAs in the endothelial function caused by ox-LDL after treatment with atorvastatin, through integrated analysis of transcriptomics data and data from lncRNA microarrays. Their study reported a list of mRNAs and lncRNAs differentially expressed after treatment with ox-LDL for 24 h; after treatment with ox-LDL followed by administration of atorvastatin; and after the administration of atorvastatin [19].

Although various lncRNAs have been described in lipid metabolism, they involve mainly functions reported in vitro. To date there is little information on the role of lncRNAs in the response to treatment with hypolipidemic drugs like statins. Considering the growing progress made in the study of the biology of lncRNAs in lipid metabolism, and the fact that these are promising findings for future use in personalised medicine, lncRNAs are significant candidates for use as predictors of response to statins. We therefore assessed the expression levels of three lncRNAs (lncRNA RP1-13D10.2; lncRNA MANTIS, also known as lncRNA n342419; lncHR1: long non-coding RNA HCV regulated 1, ENST00000549532) that have been associated with genes involved in the metabolism of cholesterol, in individuals with normal cholesterol levels and in hypercholesterolemic patients; we also assessed their value as predictors of the effectiveness of hypolipidemic treatment in THP-1 cells differentiated to monocyte macrophages and in hypercholesterolemic patients treated with atorvastatin (20 mg/day/4 weeks).

## 2. Materials and Methods

### 2.1. Selection of Subjects and Treatment Protocols 

As this was an exploratory study, the sample consisted of 20 subjects with normal cholesterol levels, selected at random, and 20 hypercholesterolemic patients of both sexes, aged between 18 and 65 years, registered at the medical centre in Chol-Chol, Temuco, Chile. The hypercholesterolemic patients were selected using the following criteria: total cholesterol levels higher than 200 mg/dL; and LDL-C higher than 140 mg/dL [20]. The hypercholesterolemic patients passed through a washout phase of 4 weeks, with indication of a low-fat diet, following the recommendations of the American Heart Association [21]. At the end of this period, the subjects with LDL-C higher than 140 mg/dL were treated with a daily dose of 20 mg of atorvastatin for 1 month (*n* = 20). 

Patients with the following conditions were excluded: familial hypercholesterolemia; liver or kidney disease; diabetes mellitus; undergoing treatment with β-blockers or diuretics; previous hypolipidemic treatment or concomitant medication that might affect the response to treatment. The patients participated in the study voluntarily and signed a written informed consent. The study protocol was approved by the Scientific Ethics Committee (CEC) of Universidad de La Frontera (No. 045_17). The investigation was carried out in accordance with the ethical principles of the Declaration of Helsinki [22]. The subjects’ clinical and demographic details were recorded, and their baseline lipid levels were measured. In the case of the hypercholesterolemic patients, the lipids profile was also determined on conclusion of the treatment. 

The blood samples were taken by routine venous puncture after fasting for 10–12 h and collected in vacutainer tubes without anticoagulant; the serum levels of TC, TG, HDL-C and VLDL were measured by routine methods [23]. The LDL-C was estimated by the Friedewald formula when the triglycerides did not exceed 400 mg/dL [24]. A blood sample treated with EDTA anticoagulant was also taken to isolate total RNA from peripheral blood leukocytes. Total RNA extraction was carried out with the mirVana^TM^ kit (Ambion, Applied Biosystems, Houston, TX, USA) following the manufacturer’s instructions. 

### 2.2. Cell Culture and Treatment

The study model used was THP-1 (Human acute monocytic leukaemia cell line, ATCC^®^ Number: TIB-202^TM^), cells of which were differentiated to monocytic (Mo) macrophages using PMA as the reagent. The THP-1 cells were kept in RPMI-1640 (Roswell Park Memorial Institute) medium supplemented with 25 mM HEPES, fetal bovine serum 10%, L-glutamine and 10,000 U/mL of penicillin-streptomycin. The cell line was cultured at 37 °C in a humidified atmosphere containing 5% of CO_2_. To differentiate THP-1 into a of Mo macrophage phenotype, the THP-1 cells (5 × 10^4^ cells/well) were seeded in 24-well plates and incubated in RPMI-1640 culture medium with 50 ng/mL of phorbol-12-myristate-13-acetate (PMA; Sigma Aldrich^®^, Darmstadt, Germany) at 37 °C for 48 h in a humidified atmosphere containing CO_2_ 5%. Activation was confirmed by evaluation of morphological changes observed under the microscope, and adhesion to the culture support. 

To separate adhered cells, the plate was rinsed twice with phosphate buffer sterile (PBS) and then incubated with Trypsin 0.05% (Invitrogen, ThermoFisher, Waltham, MA, USA) for 2 min in an incubator with humidification (37 °C and 5% CO_2_); the cells were separated completely by gentle scraping, collected, and rinsed twice with RPMI-1640 medium. After collection, cell viability was estimated at over 95% by exclusion with trypan blue. To determine the degree of differentiation, the differential expression of the genes TNF-α, IL1-β and MMP-9 were measured in monocytic THP-1 and THP-1 differentiated to Mo macrophages, using q-PCR. 

The in vitro viability was tested by cell proliferation in an aqueous solution of CellTiter 96^®^ (MTS) Promega (Madison, WI, USA) to determine the cytotoxic effect of atorvastatin on the viability of THP-1 cells differentiated to a Mo macrophage phenotype. The viability trials followed the manufacturer’s protocols. The THP-1 cells were placed in 96-well plates (1 × 10^4^ cells/well) and differentiated to Mo macrophages with PMA. Subsequently, they were exposed to different concentrations of atorvastatin (0; 2.5; 5; 10 and 20 µM) and left to culture for 24 h. At the end of the treatment time, the culture medium was removed and replaced with 20 µL of MTS reagent in each well, followed by incubation for 4 h at 37 °C. The absorbance was determined by a microplate reader (NanoQuant, Infinite^®^ M200PRO–Tecan, Redwood, CA, USA) at 490 nm. The results were expressed as the percentage viability in relation to the vehicle. 

To assess the effect of atorvastatin in vitro, the THP-1 model cell was standardised as follows: THP-1 were differentiated to Mo macrophages with 50 ng/mL of PMA for 48 h, then the Mo were incubated with different doses of atorvastatin (0; 2.5; 5; 10 and 20 µM) for 24 h. Controls were implemented using cells which were not exposed to the medication, and cells exposed only to the treatment vehicle (methanol at 0.1%). The atorvastatin (Sigma Aldrich) was dissolved in methanol. The trials were carried out in technical and biological triplicate. A suspension was generated containing 5–10 × 10^6^ cells/mL. The total RNA was extracted from cells treated with PMA and untreated cells, and subsequently from cells treated with PMA at different doses of atorvastatin using the reagent Trizol (Invitrogen, Waltham, MA, USA) and following the manufacturer’s instructions.

### 2.3. Analysis of the Expression of lncRNAs and Genes by RT-qPCR

cDNA was synthesised from 1 µg of total RNA extracted using the High-Capacity RNA-to-cDNA™ kit (Applied Biosystems, Waltham, MA, USA), following the manufacturer’s instructions. The reverse transcription reaction (20 μL) was carried out in a conventional thermocycle in two steps: 37 °C for 60 min and a final stage of 95 °C for 5 min. 

To evaluate the degree of differentiation between monocytic THP-1 cells and THP-1 differentiated to Mo macrophages, the differential expressions of the genes TNF-α, IL1-β and MMP-9 were determined by q-PCR. The expression of the lncRNAs and the genes involved in cholesterol homeostasis, both in vivo and in vitro, was quantified by real-time PCR in a 48-well plate using Fast^®^ SYBR Green Master Mix (Applied Biosystems, Foster City, CA, USA) to detect the cDNA amplification in a final volume of 20 μL. The primer sequences used are shown in Table 1. The primers for the expression of the genes RPL27, HMGCR, MMP9 and TNF-α were designed using the Primer-BLAST software. The RT-PCR data were normalised using the geometric mean of multiple internal controls: for LDLR, we tested RPS29, RPL27 and RPS13; for analysis of the lncRNAs, we tested U6 snRNA, RP11-204K16.1 and XLOC_012542 [25].

Our results showed that two genes, RPL27 and U6 snRNA, presented the most stable expression; they are therefore the optimum reference genes for analysis of genes and lncRNAs respectively. The reactions were subjected to the following thermocycle design using StepOne equipment (Applied Biosystems, Waltham, MA, USA): initial activation at 95 °C for 20 s, followed by 40 compound denaturing cycles at 95 °C for 3 s and an Annealing/Extension step at 60 °C for 30 s. The data were analysed using the comparative threshold cycle method. Negative controls and technical duplicates were implemented.

### 2.4. Statistical Analysis 

The sample size was calculated using the formula proposed by Hart et al. [30]. According to this formula, a minimum sample size of 16 subjects per group is sufficient to assess differentially expressed RNAs, with α value of 0.05, statistical power of 90% and a coefficient of variation of 0.6 to detect a 2-fold change. In consideration of possible losses of 20%, we decided to assess 20 subjects per group. Descriptive statistics were prepared for the demographic, clinical and laboratory variables. Statistical tools such as mean and standard deviation were used for the continuous variables. The lipids levels of the hypercholesterolemic individuals were analysed at baseline and post-treatment with atorvastatin; changes were summarised as percentages. 

To explore the differential behaviour of the selected lncRNAs and genes, the hypercholesterolemic individuals treated with atorvastatin were grouped into sub-groups, generating response quartiles based on the percentage reduction of LDL-C. Values before and after treatment with atorvastatin, and comparisons of the extreme quartiles in the patients, were analysed using Student’s t-test. After observation of the expression behaviour of the lncRNAs, a correlation analysis was carried out by linear regression to analyse expression of the genes associated with the pharmacodynamics of statins. 

The relative expressions of the lncRNAs and genes in the subjects with normal cholesterol levels was compared with those in the hypercholesterolemic patients by Student’s t-test. The effect of treatment with atorvastatin on the THP-1 cells differentiated to macrophages was analysed using one-way ANOVA, with Dunnett’s multiple comparison as a post test. The data obtained on the expression of the selected genes and lncRNAs were managed in Excel and then analysed with the GraphPad Prism statistics programme, version 5.0 (GraphPad Software Inc., San Diego, CA, USA). All the statistical tests applied to the hypotheses had two tails. The level of significance was set at *p* < 0.05.

## 3. Results

### 3.1. Clinical and Demographic Variables of the Study Groups

The clinical and demographic characteristics of the study subjects, both hypercholesterolemic and with normal cholesterol levels, are shown in Table 2. The mean age of the subjects with normal cholesterol was 33.70 ± 10.92, and that of the hypercholesterolemic group was 47.30 ± 11.35. There was a prevalence of females in both study groups. The clinical variables were within the reference values in all cases. In the hypercholesterolemic patients, the hepatic enzymes were normal after treatment with atorvastatin, and none of the individuals experienced adverse effects.

### 3.2. Lipid Profile of the Study Groups 

Table 3 shows the baseline lipids levels of the normal cholesterol group and the hypercholesterolemic patients in the study, as well as the levels of the hypercholesterolemic patients post-treatment with atorvastatin (20 mg/day/4 weeks). As was to be expected, the hypercholesterolemic individuals presented an atherogenic lipid profile, with higher concentrations of TC (*p* < 0.0001), LDL-C (*p* < 0.0001), VLDL (*p* = 0.0007), TG (*p* = 0.0014) and TC/HDL-C (*p* < 0.0001) than the normal cholesterol group. However, no significant differences were observed between the HDL-C values of the two groups. It was also observed that the treatment of the hypercholesterolemic patients was effective in reducing the levels of TC, LDL-C and TC/HDL-C (*p* < 0.0001). However, the treatment had no significant effect on the levels of HDL-C, VLDL and TG (*p* > 0.05). 

### 3.3. Expression of Genes and lncRNAs in the Normal Cholesterol Group and the Hypercholesterolemic Patients

**Figure 1:**
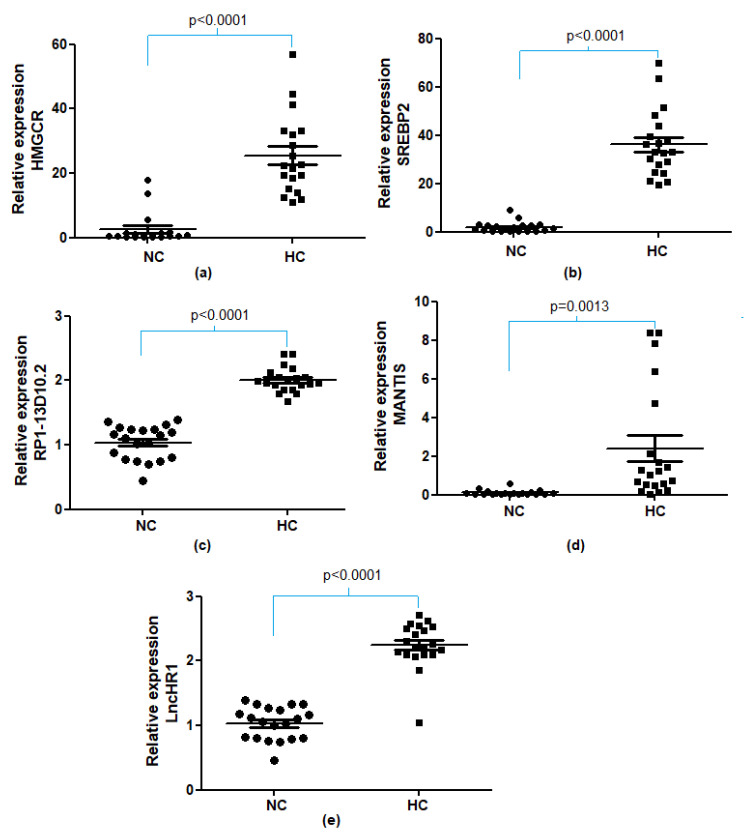
Expression of genes (**a**) HMGCR; (**b**) SREBP2 and lncRNAs (**c**) RP1-13D10.2; (**d**) MANTIS; (**e**) LncHR1 in normal cholesterol group and hypercholesterolemic patients. The relative levels were quantified using real-time PCR applied to total RNA extracted from leukocyte cells of normal cholesterol individuals (NC) and hypercholesterolemic patients (HC). *p*-value obtained by unpaired Student’s *t*-test. Data were normalised against the RPL27 reference gene for genes and the U6 reference gene for lncRNAs. The results were obtained from technical duplicates. Circles represent normocholesterolemic individuals, and squares represent hypercholesterolemic patients.

The gene expression of HMGCR (*p* < 0.0001) and SREBP2 (*p* < 0.0001), as well as expression of the lncRNAs RP1-13D10.2 (*p* < 0.0001), MANTIS (*p* = 0.0013) and lncHR1 (*p* < 0.0001), showed significant increases in the hypercholesterolemic patients as compared to the normal cholesterol individuals (Figure 1).

### 3.4. Effect of Atorvastatin on the Expression of Genes and lncRNAs in Hypercholesterolemic Patients after Treatment

**Figure 2:**
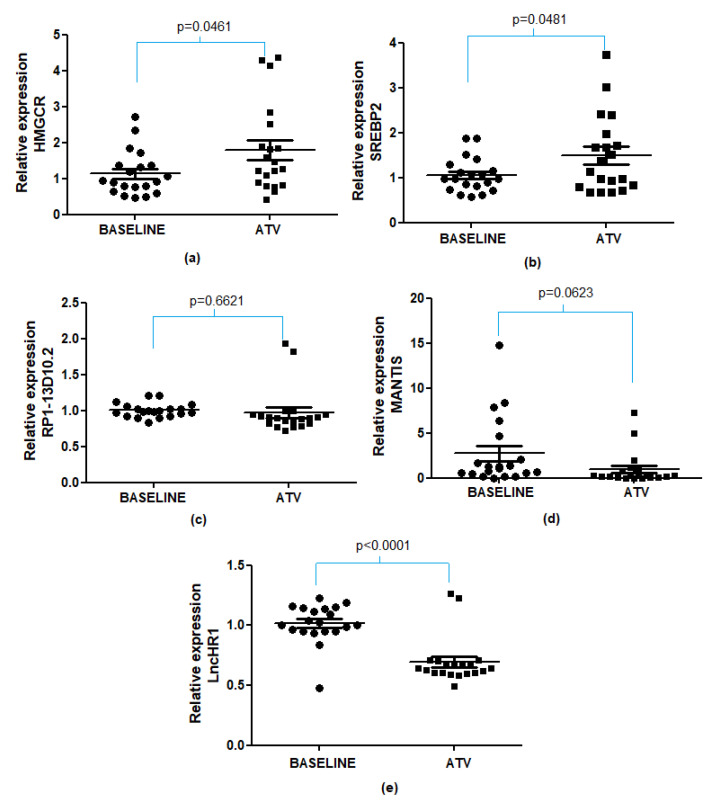
Expression of the genes (**a**) HMGCR; (**b**) SREBP2 and the lncRNAs (**c**) RP1-13D10.2; (**d**) MANTIS; (**e**) LncHR1 in hypercholesterolemic patients treated with atorvastatin. The levels of expression were measured by real-time PCR based on total RNA extracted from leukocyte cells of hypercholesterolemic patients before (baseline) and after treatment with atorvastatin (ATV, 20 mg/day/4 weeks). *p*-value obtained by paired Student’s *t*-test. Data were normalised against the RPL27 reference gene for genes and the U6 reference gene for lncRNAs. The results were obtained from technical duplicates. Circles represent hypercholesterolemic patients before treatment with atorvastatin (Baseline) and squares represent the same individuals after treatment with atorvastatin (ATV).

Treatment with atorvastatin (20 mg/day/4 weeks) had a significant positive effect in regulating gene expression of both HMGCR (*p* = 0.0461) and SREBP2 (*p* = 0.0481) in hypercholesterolemic patients when their levels before and after hypolipidemic treatment were compared (Figure 2). It may be noted that the genes studied are linked to cholesterol metabolism, and these results show that they are influenced by the action of statins. Furthermore, atorvastatin had a significant negative regulatory effect on the expression of lncHR1 (*p* < 0.0001) in hypercholesterolemic subjects treated with the drug. However, no significant differences in the lncRNAs RP1-13D10.2 (*p* = 0.6621) and MANTIS (*p* = 0.0623) were found post-treatment (Figure 2). 

**Figure 3:**
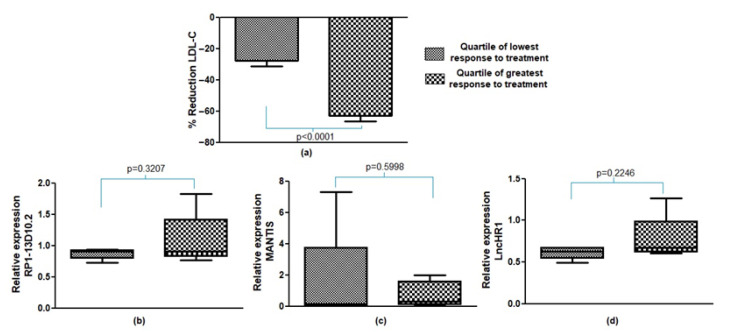
Analysis by response quartiles of hypercholesterolemic patients treated with atorvastatin. (**a**) Percentage reduction of LDL-C in the hypercholesterolemic patients treated with atorvastatin 20 mg/day/4 weeks, grouped by extreme quartiles of response to the hypolipidemic drug. The quartiles with greatest and lowest response to the treatment are shown. Relative expression of the lncRNAs in extreme quartiles: (**b**) RP1-13D10.2; (**c**) MANTIS; (**d**) LncHR1. Data were normalised against the U6 reference gene. *p*-value obtained by unpaired Student’s *t*-test.

Study of the reduction caused by hypolipidemic treatment with atorvastatin showed great variability in the response (Δ reduction max − min = 45%). For this reason, the hypolipidemic response was divided into response quartiles, grouping patients with greater or lower response to the treatment; LDL-C was used as the effectiveness variable (Figure 3a). The mean reduction for the quartile with the lowest response to atorvastatin was −27.58%, while in the quartile with the greatest response the reduction was −62.69%. The difference between the mean reductions was statistically significant (*p* < 0.0001). In contrast, study of the levels of expression between the extreme quartiles, using LDL-C as the response variable, showed no significant differences in the lncRNAs RP1-13D10.2 (*p* = 0.3207), MANTIS (*p* = 0.5998) and lncHR1 (*p* = 0.2246) (Figure 3b–d). 

**Figure 4:**
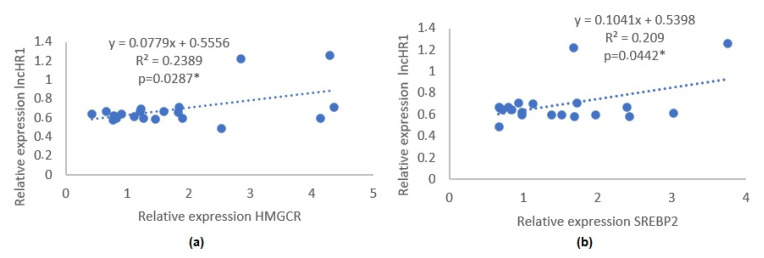
Dispersion diagrams to illustrate the correlation of expression of the genes (**a**) HMGCR and (**b**) SREBP2, associated with the pharmacodynamics of statins, and the downregulated lncRNA lncHR1 in hypercholesterolemic patients after treatment with atorvastatin 20 mg/day/4 weeks. Correlation results obtained by simple linear regression analysis. The line represents the trend line of the data obtained from the correlation of the evaluated genes. The points cor-respond to each of the gene pairs (x value and its respective y value). The asterisk (*) represents significant *p* value obtained from the analysis of variance.

After observation of the expression behaviour of lncHR1, a correlation analysis was carried out of the expression of the genes associated with the pharmacodynamics of statins (Figure 4). Linear regression analysis showed a regular positive correlation between gene over-expression of HMGCR, SREBP2 and lncHR1 in hypercholesterolemic patients treated with atorvastatin. 

### 3.5. Expression of lncRNAs and Genes In Vitro after Treatment with Atorvastatin

**Figure 5:**
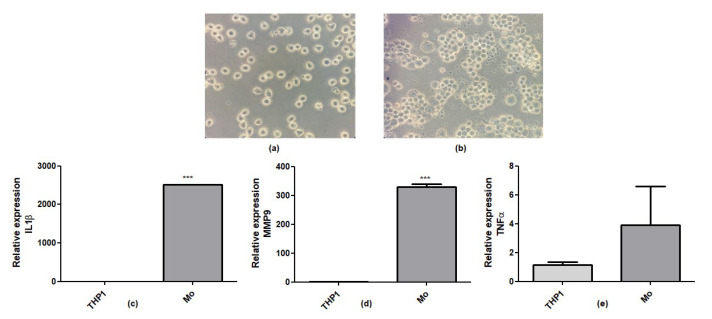
THP-1 is differentiated to Mo macrophages by using 50 ng/mL of PMA for 48 h. Morphology of (**a**) Monocytic THP-1, (**b**) THP-1 differentiated to Mo macrophage. Cell size and granularity are shown in photographs taken through an Olympus CKX41 inverted microscope (objective 40X). Gene expression of (**c**) IL1-β, (**d**) MMP-9 and (**e**) TNF-α. RT-qPCR was normalised by RPL-27 reference gene. The bars represent the mean expression for each group ± standard deviation. *p*-value obtained by Student’s *t*-test. THP-1: monocytic cells; Mo: macrophage derived from THP-1. *** *p* < 0.0001.

Exposure of THP-1 to PMA induces cell differentiation to Mo macrophages. The human leukaemia cell line (THP-1) is differentiated into cells similar to Mo macrophages after treatment with 50 ng/mL of PMA for 48 h. This results in adhesion to the culture support, which can be seen under inverted electron microscope. Mo macrophages imitate native macrophages derived from monocytes with a growing cytoplasmic volume, showing an increase in granularity (Figure 5b) compared with undifferentiated THP-1 (Figure 5a). The differentiation between THP-1 and Mo cells is also characterised by over-expression of pro-inflammatory mediators. This behaviour was evaluated by RT-qPCR, comparing expression of the genes IL1-β, MMP-9 and TNF-α. The results showed that the genes IL1-β (*p* < 0.0001) and MMP-9 (*p* = 0.0010) increased significantly after treatment with 50 ng/mL of PMA for 48 h. In contrast, the gene TNF-α (*p* = 0.4056) showed no significant differences post-treatment, however it did show a tendency to increased expression (Figure 5c–e).

**Figure 6:**
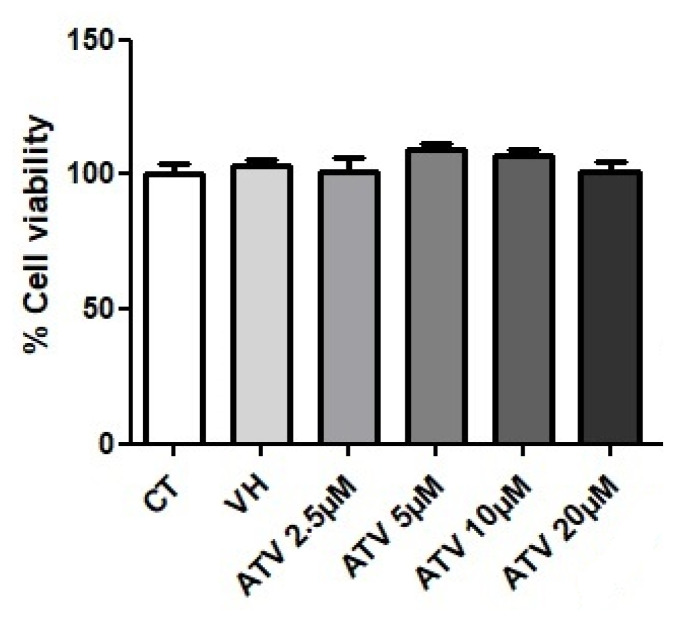
Effect of atorvastatin on the viability of THP-1 differentiated to Mo macrophages (0; 2.5; 5; 10 and 20 μM atorvastatin). Cell viability tested by MTS assay. The data are expressed as mean ± standard deviation. They were analysed statistically by ANOVA, with Dunnett’s multiple comparison as a post test. CT: control, VH: methanol vehicle, ATV: atorvastatin.

The viability of the THP-1 cell line differentiated to Mo macrophages in the presence and absence of atorvastatin during 24 h of treatment was assessed in concentrations of 0 (vehicle and control); 2.5; 5; 10 and 20 µM (Figure 6). Treatment of the cells with atorvastatin at the concentrations tested showed an acceptable range of cell viability (>90%).

**Figure 7:**
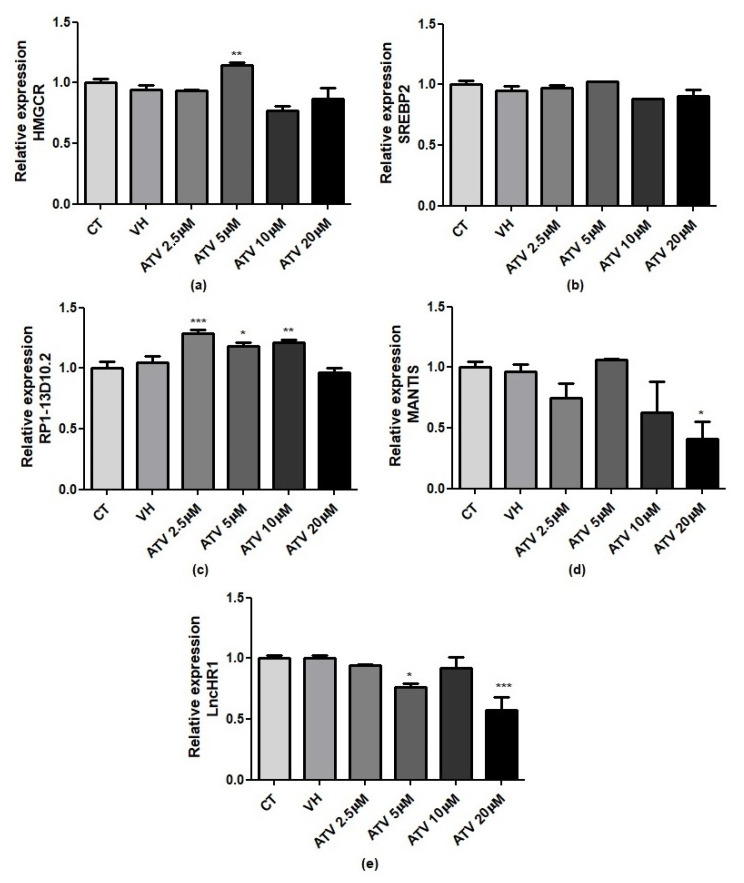
Expression of the genes (**a**) HMGCR and (**b**) SREBP2 and the lncRNAs (**c**) RP1-13D10.2, (**d**) MANTIS and (**e**) lncHR1 in THP-1 cells differentiated to Mo macrophages (0; 2.5; 5; 10 and 20 μM atorvastatin). The data are expressed as mean ± standard deviation. Data were normalised against the RPL27 reference gene for genes and the U6 reference gene for lncRNAs. They were analysed statistically by ANOVA, with Dunnett’s multiple comparison as a post-test (* *p* ≤ 0.05, ** *p* ≤ 0.001 and *** *p* ≤ 0.0001). CT: control, VH: methanol vehicle, ATV: atorvastatin.

The Figure 7 shows the effect of atorvastatin at the concentrations of 0 (control and vehicle); 2.5; 5; 10 and 20 μM for 24 h on the gene expression of HMGCR, SREBP2 and the lncRNAs in the THP-1 cell line differentiated to Mo macrophages. Treatment with atorvastatin significantly increased gene expression of HMGCR compared to the control without treatment and the vehicle control at a concentration of 5 μM. However, no significant differences were observed in the expression of the gene SREBP2 at any of the concentrations studied. All the lncRNAs showed significant differences after treatment in at least one of the concentrations tested. The lncRNA MANTIS showed a tendency to reduced expression, which was significant at the highest concentration tested (20 μM). In contrast, expression of the lncRNA RP1-13D10.2 increased significantly after treatment with atorvastatin at the concentrations studied; only at the concentration of 20 μM were no significant differences observed. In the case of lncHR1, treatment with atorvastatin reduced expression at two of the concentrations tested (5 and 20 μM). The results obtained in vitro for lncHR1 agree with those obtained in the study in vivo. No differences were observed in expression between the control without treatment and the vehicle control.

## 4. Discussion

Maintaining cholesterol homeostasis is essential for normal cell and systemic functioning. The homeostasis of cellular cholesterol is maintained by regulation of cholesterol synthesis, release, and uptake from lipoprotein transporters. In recent years, lncRNAs have been found to be important regulators of cholesterol homeostasis, and deregulation of their expression has been associated with lipids-related diseases, such as hepatic and cardiovascular diseases [31]. The present investigation assessed the levels of expression of two genes and three lncRNAs previously associated with cholesterol metabolism in people with normal cholesterol levels and hypercholesterolemic patients, to demonstrate their deregulation in lipids-related diseases. The gene expression of HMGCR and SREBP2, as well as expression of the lncRNAs RP1-13D10.2, MANTIS and lncHR1, showed significant increases in hypercholesterolemic patients as compared to the normal cholesterol individuals. 

Modulation of the homeostasis of cholesterol and fatty acids by the fatty acids present in the diet is mediated by changes in the expression of key genes involved in the metabolism of lipoproteins. A recent study evaluated the short-term effect of the ingestion of fats in the diet on gene expression in 12 healthy, non-obese men with normal plasma lipid profiles. After a fat-rich diet, the plasma concentrations of cholesterol, LDL cholesterol and HDL cholesterol were significantly higher than the concentrations observed after consumption of a low-fat diet. The high-fat diet also resulted in significant increases in the expression of messenger RNA of various key genes involved in the metabolism of lipoproteins like SREBP2 and LDLR; however, no significant differences were found in the gene expression of HMGCR [32].

Turning to the lncRNAs analysed in this study, previous studies have reported that over-expression of the lncRNA RP1-13D10.2 increases levels of LDLR transcription without affecting stability; however, RP1-13D10.2 is in chromosome 6, while LDLR is found in chromosome 19, therefore RP1-13D10.2 probably does not affect LDLR through changes in the potentiating activity of this gene. The most probable function of RP1-13D10.2 is as a guide molecule [13]. LncHR1 has hypolipidemic effects in transgenic mice fed a fat-rich diet. It inhibited the level of the protein SREBP-1c, limited the accumulation of TG and affected the metabolism of lipoproteins in the mice in the lncHR1^TG^ group [27]. With respect to MANTIS, previous studies have reported that its expression is altered in the context of disease. MANTIS was downregulated in the lungs of patients with terminal idiopathic pulmonary arterial hypertension, a disease of small vessels accompanied by endothelial dysfunction, endothelial apoptosis and proliferation [26]. Our results suggest that RP1-13D10.2, lncHR1 and MANTIS are over-expressed in hypercholesterolemic subjects, showing that it is deregulated in lipids-related diseases; this is the first study of this phenomenon in patients with this disease. These findings agree with the reports of other authors on the role played by these lncRNAs in the metabolism of lipids. 

Few studies have been carried out to date with lncRNAs in the context of treatment with statins. The effect of statins is mediated by a strict process of transcription regulation of genes involved in lipid biosynthesis, such as LDLR, HMGCR and SREBP2. Various studies have shown that statins are able to modulate the expression of genes not only in terms of the hypolipidemic effect, but also their anti-inflammatory and anti-atherogenic impacts. These effects support the use of this drug as a highly effective treatment to reduce the risk of cardiovascular disease not only in hypercholesterolemic patients, but also those with normal cholesterol levels suffering diabetes or with a history of coronary events [33]. In the group of hypercholesterolemic patients, atorvastatin was effective in optimising the lipids profile, reducing levels of total cholesterol and LDL-C; however, it did not produce changes in levels of HDL-C and TG. In the distribution of the response to hypolipidemic treatment with atorvastatin obtained when LDL-C is used as the effectiveness variable, hypercholesterolemic patients presented reductions in a range of −22% to −67%, supporting the fact that atorvastatin is considered one of the most powerful statins in the market, since it is used in treatments which seek to achieve reductions of between 30 and 35% with doses of 10 mg [34,35].

In the present study we also assessed the levels of expression of these lncRNAs and the genes HMGCR and SREBP2 in hypercholesterolemic patients after treatment with atorvastatin. We observed that atorvastatin increased the expression of the genes HMGCR and SREBP2 in the patient’s post-treatment. Likewise, increased expression of HMGCR has been reported in peripheral blood mononuclear cells (PBMC) [36] after treatment with atorvastatin, which is consistent with the action mechanism of the drug. Atorvastatin has also been shown to have a regulatory effect on gene expression of SREBP and SCAP, as molecules directly linked with the transcription regulation of LDLR [37]. On the other hand, a different study reported an insignificant effect of atorvastatin on the levels of expression of mRNA HMGCR in mononuclear cells in the circulation of subjects treated with atorvastatin, 20 mg/day for 4 weeks [38], like the treatment applied in our study.

Regarding the relationship between lncRNAs and the genes evaluated in the present investigation, previous studies have elucidated the potential mechanism by the lncRNAs regulate cholesterol metabolism, through the evaluation of the expression of cholesterol-related genes, and among them are the analysed genes in this study. For example, lncARSR modulates hepatic cholesterol biosynthesis in vivo through increased gene expression of SREBP-2, HMG-CoA reductase (HMGCR), HMG-CoA synthase (HMGCS) and squalene synthase (SQS). Similarly, over-expression of lncARSR also increased HMGCR mRNA and protein levels in vitro. Authors concluded that the potential mechanism by which lncARSR regulates HMGCR expression is through expression of the primary transcription factor SREBP-2. SREBP-2 gene expression was increased both in vitro and in vivo. These results indicate that lncARSR can regulate HMGCR expression through SREBP-2 [28]. 

For the lncRNA LASER, some key genes involved in cellular cholesterol metabolism (SREBP1\SREBP2\apoB100\HMGCR\FAS\VLDLR\LDLRP\HNF1α\PCSK9\PPARG) were also evaluated after LASER knockdown. However, only the HNF-1α\PCSK-9\PPARG genes changed their expression after LASER downregulation [14]. Turning the lncRNAs evaluated in the present investigation, previous studies have related lncHR1 with the activity of the SREBP-1c gene. To confirm the effect of lncHR1 on SREBP-1c, authors transfected Huh7 cells with a lncHR1 over-expression construct. The expression of lncHR1 increased in a dose-dependent manner. In contrast, SREBP-1c mRNA gradually decreased. Thus, lncHR1 has been described as a negative regulator of SREBP-1c. However, the study concludes that the molecular mechanism of lncHR1 regulation and SREBP-1c expression needs further elucidation [27]. There are no previous studies relating this lncRNA with the evaluated genes in the present investigation. Of the evaluated three lncRNAs, only lncHR1 was significantly affected in hypercholesterolemic patients after treatment with atorvastatin; further it was positively correlated with the genes, which suggests that these genes could be influencing in the regulation of this lncRNA. However, the molecular mechanism that describes this relationship is still unknown, and further studies are necessary.

In the case of RP1-13D10.2, a previous study quantified the expression of some genes involved in cholesterol biosynthesis (HMGCR, HMGCS1) and cholesterol uptake (LDLR, MYLIP and PCSK9). However, over-expression of RP1-13D10.2 only increased LDLR transcripts [13]. Similarly, MANTIS has been related to the adhesion molecule ICAM-1 and the transcription factors KLF2 and KLF4 [15]. However, there are no previous studies that relate it with the cholesterol metabolism genes evaluated in the present investigation.

Various authors have also examined the effects of statins on the expression of other genes that play an important role in cholesterol metabolism. For example, reduced expression of the transporter genes ABCA1 and ABCG1 was found in the peripheral blood mononuclear cells (PBMC) of individuals with primary (non-familial) hypercholesterolemia treated with statins (atorvastatin and ezetimibe + simvastatin in doses of 10 mg/day/4 weeks for each group) [39]. An interesting recent review summarises the association between lncRNAs and the roles of ABCA1 and ABCG1. It has been reported that ABCA1 is regulated by different lncRNAs such as MeXis, GAS5, TUG1, MEG3, MALAT1, Lnc-HC, RP5-833A20.1, LOXL1-AS1, CHROME, DAPK1-IT1, SIRT1 AS, DYNLRB2-2, DANCR, LeXis, LOC286367, and LncOR13C9. Furthermore, ABCG1 is regulated by the lncRNAs TUG1, GAS5, RP5-833A20.1, DYNLRB2-2, ENST00000602558.1, and AC096664.3 [40]. All these reports show the potential of lncRNAs to regulate genes associated with the metabolism of cholesterol, and their influence on the development of atherosclerosis. 

In the present investigation, we observed that atorvastatin reduced the expression of lncHR1 in hypercholesterolemic subjects treated with the drug. However, no significant differences in the lncRNAs RP1-13D10.2 and MANTIS were found post-treatment. Few studies have been done to date on the lncRNAs analysed in this investigation. Previous studies relate lncHR1 with regulation of the metabolism of hepatic lipids by SREBP-1c inhibition. In vivo data from transgenic mice showed that individuals with expression of lncHR1 presented less hepatic expression of SREBP-1c, FAS, acetyl-CoA carboxilase α (ACCα) and less hepatic and plasma TG after feeding with a high-fat diet [27]. A subsequent study showed that lncHR1 inhibits SREBP-1c levels through phosphorylation of the PDK1/AKT/FoxO1 pathway [41]. Our results show reduced expression of lncHR1 in hypercholesterolemic patients after treatment with atorvastatin. Furthermore, we obtained a regular positive correlation between this lncRNA and over-expression of the genes HMGCR and SREBP2. These findings highlight the potential role of this long non-coding RNA as a determinant of interindividual variation in response to this drug.

In contrast, the lncRNAs RP1-13D10.2 and MANTIS showed no significant differences post-treatment with atorvastatin in the present study. These results differ from those reported by other authors. A study carried out using simvastatin and sham-incubated lymphoblastoid cell lines from patients with genotypes rs6924995 previously imputed, found that statin-induced change in RP1-13D10.2 levels differed between the cell lines of Caucasian and Afro-American LDL-C response distributions. Over-expression of RP1-13D10.2 in Huh7 and HepG2 increased LDLR transcription levels, increased LDL uptake, and decreased mean levels of APOB. The study concluded that RP1-13D10.2 regulates LDLR and may contribute to low-density lipoprotein cholesterol response to statin treatment [12]. Another recent study showed that laminar flow, like statins, induces the lncRNA MANTIS through a KLF2/KLF4-dependent mechanism. The study found that MANTIS mediates various positive aspects of the signalling induced by flow and statins, and has protective endothelial effects. The inhibiting effect of MANTIS on ICAM-1 was observed in vitro in endothelial cells, and in vivo in humans. MANTIS expression was higher in the stable plates of the carotid artery of patients treated with statins than in similar samples from patients who had not received statins. Overall, the protective effects of laminar flow and statins is attributed, at least in part, to MANTIS. Given that the induction of MANTIS imitates the beneficial effects of statins on the endothelial function, the study proposes that strategies to increase MANTIS could improve vascular function in patients who do not respond to treatment with statins [15]. Our results for the lncRNAs RP1-13D10.2 and MANTIS, and for the response to statins, offer a first approach to the role played by these lncRNAs in the treatment of hypercholesterolemic patients with atorvastatin. The differences between our results and those obtained by other studies are due principally to the types of samples used and the treatments applied. 

The in vitro results reported in the present study were obtained by means of a widely used atherosclerosis cell model, the THP-1 cell line, which consists of monocyte human cells derived from acute monocyte leukaemia [42,43,44,45]. The protocol used in this investigation to transform the THP-1 cells into adhesive macrophages was based on standardisations developed previously by our research group, and on a literature review [29,46]. Treatment of THP-1 with PMA induced the primary formation of Mo macrophages, with a specific phenotype, shown in the cell morphology by increased cell volume and adhesion to the culture support [47]. As reported by other authors [29,48], this differentiation can be confirmed by the expression of pro-inflammatory cytokines like IL1-β and the matrix metalloproteinase MMP-9, which showed a significant increase in the Mo macrophage stimulated by PMA as compared with the unstimulated monocytic THP-1. 

After activation to macrophages, the cells were subjected to different doses of atorvastatin for 24 h and the level of expression of the mRNA of HMGCR and SREBP2 were assessed as predictors of hypolipidemic response, as well as the levels of expression of the lncRNAs. HMGCR presented increased expression in the cells treated with atorvastatin at the concentration of 5 μM as compared with the control without treatment and the vehicle control. SREBP2 expression, in contrast, showed no significant differences at any of the concentrations studied. Likewise, a pilot study showed that different statins produce highly divergent changes in the gene expression profiles in HepG2 cells. Relative quantification of HMGR revealed an increase in levels of mRNA for that gene in the cells treated with statins (atorvastatin, fluvastatin and simvastatin) in comparison with the cells treated with buffer. The most prominent change was found in the cells cultured with atorvastatin, while the change in the expression of mRNA induced by fluvastatin was significantly smaller [49]. 

In vitro, however, all the lncRNAs showed significant differences in expression after treatment with atorvastatin in at least one of the concentrations tested. The lncRNA MANTIS showed a tendency to reduced expression, which was significant at the highest concentration tested. In contrast, expression of the lncRNA RP1-13D10.2 increased significantly after treatment with atorvastatin at the concentrations studied. In the case of lncHR1, treatment with atorvastatin reduced expression at two of the concentrations tested. Increased expression of lncHR1 in Huh7 cells infected with the hepatitis C virus (HCV) has also been reported. This over-expression suppressed the activity of SREBP-1c and FAS in vitro. Its presence was confirmed by RT-qPCR in 11 cell lines. The authors posited that lncHR1 could regulate the phosphorylation of AKT/FoxO1 [27]. Similarly, RP1-13D10.2 expression has been reported in immortalised lymphoblastoid cell lines derived from participants in a clinical trial after exposure in vitro to simvastatin 2 μM. The expression was detected both in the cells treated with statin and in the sham cells. Transitory transfection with a plasmid that over-expresses the gene RP1-13D10.2 was applied to Huh7 cells for 48 h; the over-expression of RP1-13D10.2 was confirmed by qPCR and the gene expression of LDLR was affected. Similar effects were observed of over-expression of RP1-13D10.2 in the human hepatoma cell line HepG2. The levels of expression of RP1-13D10.2 were significantly reduced after elimination of SREBF2 in Huh7 cells. The study found evidence that RP1-13D10.2 is regulated by LXR, since incubation of HepG2 with a LXR agonist increased the expression levels of this lncRNA [13]. Elsewhere it is reported that MANTIS is induced by statins. Curiously, the statins cerivastatin, fluvastatin, simvastatin and atorvastatin induced the expression not only of KLF2 and KLF4, but also of MANTIS in HUVEC and HAoEC. Furthermore, the elimination of KLF2 or KLF4, mediated by siRNA, reduced the statin-induced expression of MANTIS [15]. Our in vitro results on the effect of the hypolipidemic atorvastatin in the expression of these lncRNAs are pioneering in this area.

Finally, our data indicate that lncHR1 is deregulated both in vivo and in vitro assays. This lncRNA was described for the first time by Li and colleagues. LncHR1 (ENST00000549532) is only one transcript from gene ENSG00000257400, which is contains 420 bp. RNA in situ hybridisation in Huh7 cells showed lncHR1 was concentrated in the nucleus and cytoplasm. LncHR1 is an HCV-related lncRNA and likely protects host cells from dysfunctional lipid synthesis. This lncRNA negatively regulates endogenous SREBP-1c and FAS; and it can suppress TG accumulation and may be a protective molecule against hepatic steatosis. TG and LD accumulation can be inhibited by lncHR1 in both cultured hepatoma cells and in transgenic mice. Authors suggest that lncHR1 may serve as a new molecule for the treatment of TG accumulation in liver, and may be a protective host non-coding RNA, which can be up regulated in some diseases and avoids improper activation of the lipid synthesis pathway. Thus, lncHR1 affects cellular lipid metabolism by regulating critical lipogenic transcriptional factors SREBP-1c [27]. On the other hand, LncHR1 regulate SREBP-1c levels and the phosphorylation of AKT in the steatosis cell model. Detailed molecular mechanisms mediated by lncHR1 are associated with the phosphorylation AKT/FoxO1 in Huh7 cell lines. Simultaneously, lncHR1 affect the location of FoxO1 inside and outside of the nucleus. Furthermore, the phosphorylation of PDK1 upstream of AKT is regulated through over-expression or knockdown lncHR1. In summary, lncHR1 inhibits SREBP-1c levels through the phosphorylation of the PDK1/AKT/FoxO1 axis [41]. Our results suggest that lncHR1 is also regulated by the HMGCR and SREBP2 genes, further confirming its role in cholesterol metabolism.

Finally, there are various limitations to the results obtained in the present work. Firstly, the restricted quantity of lncRNAs selected for the expression studies in vitro and in vivo prevented us from obtaining a clearer view of the impact of hypolipidemic treatment on these molecules. Although we successfully found a deregulation effect associated with atorvastatin treatment, we cannot discount the possibility that other lncRNAs may be similarly affected. Secondly, although THP-1 cells are a suitable and commonly used model for the study of macrophages in vitro, the data obtained suggest that the alteration in the expression of lncRNAs may be specific to the cell model used; future studies should therefore include other cell models. 

In conclusion, our research shows that atorvastatin differentially modulates the expression of the lncRNAs RP1-13D10.2, MANTIS and lncHR1, associated with genes involved in cholesterol metabolism in vitro. Additionally, we highlight lncHR1 whose expression also varied in vivo. Our findings illustrate the potential of lncRNAs as determiners of variability in the response to statins, so further studies are necessary to elucidate its mechanism of action.

## Figures and Tables

**Table 1:** Primers used for RT-qPCR.

Name	Forward Primer (5′-3′)	Reverse Primer (5′-3′)	References
RP1-13D10.2	TGTGGCTCTATCACCCTCAA	AGGATGATTCGGAACACAGC	[13]
MANTIS	AACTCCTGCTCCAAACTCACTC	CCAGAGACTTTCCATTCTGATG	[15,26]
lncHR1	TTCCTGGAACTGACTGGACTTCA	GCCTGAGCAAATCAATGGATGT	[27]
U6	CTCGCTTCGGCAGCACATATAC	GGAACGCTTCACGAATTTGC	[25]
HMGCR	GCAGGACCCCTTTGCTTAGA	GCACCTCCACCAAGACCTAT	-
RPL27	TCCGGACGCAAAGCTGTCATC	GGTCAATTCCAGCCACCAGAGCAT	-
SREBP-2	CCAAAGAAGGAGAGAGGCGG	CGCCAGACTTGTGCATCTTG	[28]
IL1β	TGAAGCTGATGGCCCTAAACA	GTGGTGGTCGGAGATTCGTA	[29]
MMP9	TTCTGCCCGGACCAAGGATA	ACATAGGGTACATGACGCCC	-
TNFα	TGTAGGCCCCAGTGAGTTCT	GCAACAAGACCACCACTTCG	-

lncRNA MANTIS, also known as lncRNA n342419; lncHR1 (ENST00000549532), long non-coding RNA HCV regulated 1; U6, U6 small nucleus RNA; HMGCR, 3-hydroxy-3-methyl-glutaryl-CoA reductase gene; RPL27, ribosomal protein L27; SREBP-2, sterol regulatory element-binding protein 2; IL1β, Interleukin 1β; MMP9, matrix metalloproteinase 9; TNFα, tumoral necrosis factor α.

**Table 2:** Baseline clinical and demographic characteristics of the study groups.

Parameters	Normal Cholesterol Group (*n* = 20)	Hypercholesterolemic Group (*n* = 20)
Age (years)	33.70 ± 10.92	47.30 ± 11.35
Sex: M/F (*n*)	(2/18)	(6/14)
Glycemia (mg/dL)	86.23 ± 7.45	95.94 ± 6.94
AST/GOT (UI/L)	22.00 ± 4.04	23.73 ± 5.32
ALT/GPT (UI/L)	25.83 ± 6.12	30.25 ± 7.14
CK (UI/L)	93.14 ± 33.97	110.18 ± 25.99
Uraemia (mg/dL)	26.17 ± 8.64	30.88 ± 6.31
Blood urea nitrogen (mg/dL)	12.23 ± 4.05	13.31 ± 3.18
Blood creatinine (mg/dL)	1.01 ± 0.17	1.08 ± 0.16
Haemoglobin (Hb) (g/dL)	13.21 ± 0.90	13.83 ± 1.15
Haematocrit (Hto.) (%)	39.29 ± 2.53	41.25 ± 3.04
Leukocyte count (×103/μL)	7.40 ± 1.25	6.68 ± 1.89
Platelet count (×103/μL)	257.53 ± 58.48	243.56 ± 44.06
Total bilirubin (TB) (mg/dL)	0.65 ± 0.29	0.52 ± 0.14
Direct bilirubin (DB) (mg/dL)	0.19 ± 0.07	0.13 ± 0.04
Indirect bilirubin (IB) (mg/dL)	0.47 ± 0.24	0.37 ± 0.14

Values expressed as mean ± standard deviation. *n*, number of subjects; AST/GOT aspartate aminotransferase; ALT/GPT, alanine aminotransferase; CK, creatine kinase.

**Table 3:** Serum lipid levels of normal cholesterol subjects (baseline) and hypercholesterolemic patients (baseline and post-treatment with atorvastatin 20 mg/day/4 weeks).

Lipids (mg/dL)		Normal Cholesterol Group (*n* = 20)	Hypercholesterolemic Group (*n* = 20)	*p*-Value
TC	baseline	158.29 ± 20.49	239.35 ± 28.28	<0.0001
	treatment	---	158.15 ± 33.41	<0.0001
HDL-C	baseline	46.82 ± 6.78	44.45 ± 10.09	0.4158
	treatment	---	41.20 ± 9.48	0.3005
LDL-C	baseline	93.86 ± 17.54	164.62 ± 26.32	<0.0001
	treatment	---	91.37 ± 28.28	<0.0001
VLDL	baseline	18.19 ± 5.28	30.78 ± 13.04	0.0007
	treatment	---	24.19 ± 11.23	0.0948
TG	baseline	90.94 ± 26.38	150.40 ± 66.23	0.0014
	treatment	---	121.65 ± 55.40	0.1447
TC/HDL-C	baseline	3.51 ± 0.60	5.55 ± 0.95	<0.0001
	treatment	---	3.92 ± 0.80	<0.0001

Results expressed as mean ± standard deviation. *p*-value obtained by Student’s *t*-test. TC, total cholesterol; HDL-C, high-density lipoprotein cholesterol; LDL-C, low-density lipoprotein cholesterol; VLDL, very low-density lipoprotein; TG, triglycerides; TC/HDL-C, ratio between total cholesterol and high-density lipoprotein cholesterol.

## Data Availability

Not applicable.
